# Limited evidence of C_4_ plant consumption in mound building *Macrotermes* termites from savanna woodland chimpanzee sites

**DOI:** 10.1371/journal.pone.0244685

**Published:** 2021-02-10

**Authors:** Seth Phillips, Rudolf H. Scheffrahn, Alex Piel, Fiona Stewart, Anthony Agbor, Gregory Brazzola, Alexander Tickle, Volker Sommer, Paula Dieguez, Erin G. Wessling, Mimi Arandjelovic, Hjalmar Kühl, Christophe Boesch, Vicky M. Oelze

**Affiliations:** 1 Anthropology Department, University of California Santa Cruz, Santa Cruz, California, United States of America; 2 Fort Lauderdale Research & Education Center, Davie, Florida, United States of America; 3 Department of Anthropology, University College London, London, United Kingdom; 4 School of Biological and Environmental Sciences, Liverpool John Moores University, Liverpool, United Kingdom; 5 Department of Primatology, Max Planck Institute for Evolutionary Anthropology, Leipzig, Germany; 6 Gashaka Primate Project, Serti, Taraba, Nigeria; 7 Department of Human Evolutionary Biology, Harvard University, Cambridge, Massachusetts, United States of America; 8 German Centre for Integrative Biodiversity Research (iDiv) Halle-Jena-Leipzig, Leipzig, Germany; Southern Cross University, AUSTRALIA

## Abstract

Stable isotope analysis is an increasingly used molecular tool to reconstruct the diet and ecology of elusive primates such as unhabituated chimpanzees. The consumption of C_4_ plant feeding termites by chimpanzees may partly explain the relatively high carbon isotope values reported for some chimpanzee communities. However, the modest availability of termite isotope data as well as the diversity and cryptic ecology of termites potentially consumed by chimpanzees obscures our ability to assess the plausibility of these termites as a C_4_ resource. Here we report the carbon and nitrogen isotope values from 79 *Macrotermes* termite samples from six savanna woodland chimpanzee research sites across equatorial Africa. Using mixing models, we estimated the proportion of *Macrotermes* C_4_ plant consumption across savanna woodland sites. Additionally, we tested for isotopic differences between termite colonies in different vegetation types and between the social castes within the same colony in a subset of 47 samples from 12 mounds. We found that *Macrotermes* carbon isotope values were indistinguishable from those of C_3_ plants. Only 5 to 15% of *Macrotermes* diets were comprised of C_4_ plants across sites, suggesting that they cannot be considered a C_4_ food resource substantially influencing the isotope signatures of consumers. In the *Macrotermes* subsample, vegetation type and caste were significantly correlated with termite carbon values, but not with nitrogen isotope values. Large *Macrotermes* soldiers, preferentially consumed by chimpanzees, had comparably low carbon isotope values relative to other termite castes. We conclude that *Macrotermes* consumption is unlikely to result in high carbon isotope values in either extant chimpanzees or fossil hominins.

## Introduction

Our understanding of wild chimpanzee (*Pan troglodytes*) feeding ecology has been primarily informed by direct observations of feeding behavior within the limited number of chimpanzee communities consistently monitored by long-term research projects (e.g. [1, 2]). This bias towards a small number of chimpanzee communities has been tempered by the increasing use of indirect methods, such as stable isotope analysis, that enable large-scale cross site comparisons of the various feeding behaviors of both habituated and unhabituated chimpanzee communities [3–10]. Insights obtained from such studies are only as good as our understanding of the various stable isotope ratios, such as carbon (δ^13^C) and nitrogen (δ^15^N), of consumed organisms by chimpanzees at different locales [3–5, 11–13]. Chimpanzee δ^13^C values, from both habituated and unhabituated communities, thus far corroborate observational data that indicate chimpanzees primarily feed on vegetation relying on the C_3_ photosynthetic pathway as well as organisms consuming such vegetation [3–8, 14–17]. Still, small variation in δ^13^C values within the observed range of chimpanzee values may be representative of differences in feeding behavior across communities or across demographic classifications within communities [3–5, 8, 14]. For example, some savanna woodland chimpanzee communities exhibit δ^13^C values that are higher than baseline C_3_ vegetation even if they are still consistent with a predominately C_3_ based diet [3, 4, 8, 15]. The consumption of C_4_ plant reliant termites has been posited as a potential contributor to relatively high δ^13^C values in some savanna woodland chimpanzee communities [3].

Termites contribute key nutrients to primate diets [18, 19] and even termite soil is particularly rich in nutrients [20–23]. Across Africa many, yet far from all, chimpanzee populations have been observed to forage for termites [24] with a common preference for the large, mound-building and fungus-growing termites of the genus *Macrotermes* (*Macrotermitinae*) (summarized in [25–29]). The ecology of *Macrotermes*, and termites in general, is often cryptic and difficult to observe under natural conditions [30]. Additionally, many behaviors within the genus *Macrotermes* [27, 31–33] may vary significantly intraspecifically depending on the ecological context. *Macrotermes* is a genus of termites found exclusively in the Old World tropics. Some species, such as *M*. *bellicosus and M*. *subhyalinus*, are broadly distributed across the African continent [31, 34]. In general, *Macrotermes* spp. build large epigeal mounds and spend most of their time in the subterranean chambers and galleries throughout the year. *Macrotermes* workers forage for cellulosic debris such as leaf litter, dead grass, woody litter, and wood [35] including live crop plants [36]. Foraged items are returned to the nest as nutritional substrate for the growth of *Termitiomyces* fungus combs that consists of fungal biomass and partly decayed plant matter [37–40]. Older workers primarily feed on the fungus combs and the soldier castes rely on the workers to feed them directly with pieces of these fungal combs. By contrast, younger workers may subsist primarily on foraged plant matter as well as on the protein-rich *Termitiomyces* nodules to some extent [37]. Though primatologists have begun to account for the specific isotopic values of chimpanzee plant foods within their environments [3–5, 11–13] in cross site comparisons, there remains a need for complimentary data on insect food sources, such as *Macrotermes*, given their relevance to the diets of several chimpanzee communities (summarized in [25]).

The isotopic values of *Macrotermes* within savanna woodland ecosystems may be of specific utility in elucidating the source of comparably high δ^13^C values, particularly found in some savanna chimpanzee communities. In the unhabituated eastern chimpanzees (*P*. *t*. *schweinfurthii*) at the field site of Issa, Tanzania, van Casteren and colleagues [8] reported higher δ^13^C values than cannot be explained by the consumption of the sampled C_3_ plant foods alone. Relatively high δ^13^C fractionation factors (Δ^13^C) between chimpanzees and a selection of C_3_ plants suggested that C_4_ plants or open canopy C_3_ plant foods could be potential contributors to these chimpanzees’ diet and hence δ^13^C values. However, given that termite consumption is well documented in this population [41], the authors also posited that C_4_ plant harvesting termites may contribute to δ^13^C value enrichment in the chimpanzees. Wessling and colleagues [4] reported even higher Δ^13^C values in five western chimpanzee (*P*. *t*. *verus*) communities at the very edge of the species range in Senegal [4]. Four of the five communities in that study are unhabituated and the source of these high Δ^13^C values remained largely unclear. In the habituated community of Fongoli in Senegal however, chimpanzees have been observed to occasionally consume C_4_ plants [42]. Additionally, Fongoli chimpanzees are exceptional with regards to the frequency and intensity that they consume *Macrotermes* [43]. This consistent consumption of an organism that may rely on C_4_ vegetation is another possible explanation for the relatively high δ^13^C values observed in Senegalese chimpanzees [3, 4]. Though parsimony would suggest unhabituated chimpanzees in Senegal consume *Macrotermes*, the extent to which they do, if at all, is not yet known. Only in the Kayan chimpanzees were termites from the genus *Macrotermes* identified in feces [3]. Still, the degree to which termite consumption could contribute towards high δ^13^C values in savanna chimpanzees is not as of yet clear due to the limited dataset available on the stable isotope ratios of this termite genus within sub Saharan Africa.

Analysis of the isotopic signature of *Macrotermes* from a range of sub Saharan African sites may also have implications for paleodietary analysis of hominins. The genus *Macrotermes* diversified 6–23 million years ago as savannas spread across the African continent and remained relatively unchanged today despite climactic shifts [44, 45]. Thus, it is likely that *Macrotermes* and early hominins coexisted in the African savanna landscapes. We can further hypothesize that these termites would have had similar diets as we see in extant *Macrotermes* diets from African savanna woodlands today. Hominins in east, south, and central Africa began to consume foods enriched in ^13^C approximately 3.5 million years ago [46–57]. In relation to the high δ^13^C signatures observed in *Paranthropus robustus* and *Australopithecus africanu*s specimen from Sterkfontein, modern δ^13^C values of termites and sedges from nearby Kruger National Park were analyzed to investigate the hypothesis that either sedges or termites may be account for the observed high δ^13^C values in hominins [52]. Accordingly, termite taxa across the park had an average δ^13^C value of -20.1*‰*, with a mean of -15.3*‰* (n = 10) for open environment termites and a mean of -21.7*‰* (n = 30) for termites from closed environments. Based on these results, the authors concluded that termites could reveal a C_4_ plant dependent isotopic signature, yet their consumption could not solely account for the enriched δ^13^C values detected in the compared hominins. However, this study did not report the taxonomic classification of the termite specimen sampled (excepting a brief reference to the harvester termite genera *Trinervitermes* and *Hodotermes*) that would provide insights into whether these species could have been subject to hominin predation, nor was further ecological information on sampling locations provided.

There are a handful of other studies examining the relative contributions of C_3_ and C_4_ resources to termite diets in sub-Saharan Africa. The first systematic study of termite foraging ecology utilizing stable isotope analysis investigated the relative dependence of *M*. *michaelseni* on herbaceous (C_4_) vegetation versus woody (C_3_) vegetation by sampling from the termite head tissue and using a mixed modeling approach [58]. Both woody and herbaceous food sources contributed to the diets of *M*. *michaelseni* at two Kenyan savanna grassland sites but varied in their relative contributions. The dietary contribution from herbaceous vegetation utilizing the C_4_ photosynthetic pathway was estimated to be 70% at one site and 36% at another site. These results indicate that termites of the same species can vary significantly with regard to δ^13^C values in two, ecologically similar yet geographically distinct, environments. Additionally, this study provides some preliminary support for the hypothesis of *Macrotermes* as a partial C_4_ resource for chimpanzees as well as hominins.

A similar termite isotope study at a humid savanna site (defined as grass savanna, shrub savanna and semideciduous plateau forest) in Côte d’Ivoire also found that the relative contributions of C_3_ versus C_4_ plants of four sympatric termite species within the termite subfamily *Macrotermitinae* was considerably varied even among the same species depending on habitat type and seasonality of sampling effort [59]. More recently, Vesala and colleagues [40] investigated the isotopic values between termite castes within four *Macrotermes* colonies located in southern Kenya. While three of the four colonies exhibited δ^13^C values in range with herbaceous vegetation, one mound with more abundant grass surrounding had relatively enriched δ^13^C values. Additionally, the authors reported significant differences in the δ^13^C and δ^15^N values between castes of the same colony suggesting that the nutritional contribution of fungal symbiont (*Termitomyces*) varies between castes within the same colony [40]. These studies demonstrate the utility of adding to the African termite isotope database while distinguishing between taxa, habitat type, seasonality, as well as caste (see also [60–62]).

Termite-fishing broadly describes a behavior in which a chimpanzee inserts a vegetative tool into a passageway at the surface of a termite mound to consume the soldier termites that bite the tool. In some habitats, chimpanzees preferentially forage on *Macrotermes* during the onset of the rainy season, which may be due to an increase in accessibility during the colony’s reproductive cycle [63]. By contrast, other communities reliably consume *Macrotermes* year-round, which may be attributable to more sophisticated tool-sets [18, 64–66] or a dependence on the termites as a source of protein [43].

In the present study we analyze *Macrotermes* spp. samples collected at six chimpanzee savanna woodland field sites across equatorial Africa in the interest of further elucidating possible contributions of this genus of termites in chimpanzee isotope signatures. We focus here on *Macrotermes* spp. termites due to their status as the most commonly consumed genera among chimpanzees that termite-fish [24–27]. However, it is worth noting here that some populations do not termite-fish despite the presence of mound building termites, such as at the site of Gashaka, in Nigeria, that we report on here [67, 68]. With this study we seek to address the following two questions:

- Do we find evidence for substantial C_4_ plant consumption by *Macrotermes* across chimpanzee field sites via stable isotope analysis?
- Do we find intra-specific (between colonies) and intra-colony (between castes) isotopic variation in *Macrotermes* from the same field site?

## Material and methods

### Sample collection and isotope analysis

In this study we collected *Macrotermes* from six savanna woodland sites across Africa, that represent relatively dry and open environments inhabited by chimpanzees today. Savanna woodland habitats are more likely to have substantial amounts of C_4_ vegetation termites may rely on, as compared to forest habitats. We opportunistically collected 39 termite samples from fungus-growing mound builders at five savanna woodland chimpanzee field sites in West Africa (see Table 1 for further details and season of sampling), following a standardized sample and data collection protocol within the framework of the “Pan African Program—The Cultured Chimpanzee” project [69–71]. Permissions to conduct research were issued under the research permits N^o^ NPL/GEN/378/V/504 (*Ministère de l‘Ecologie et de la Protection de la Nature*, *Direction des Eaux*, *Forêts*, *Chasses et de la Conservation des Sols*, Nigeria), N^o^ 078/2015/OGIPAR/MEEF/Ck (*Ministère de l’Environment*, *Eaux et Forêts*, *Office Guinéen des Parks et Reserves*, Guinea), N^o^ 01316/DEF/DFG (*Direction des Eaux*, *Forêts*, *Chasses et de la Conservation des Sols*, Senegal) and N^o^ 219/MESRS/DGRSIT/TM (*Ministère de l’Enseignement Superior et de la Recherche Scientifique*, *Direction Generale de la Recherche Scientifique de de l’Inovation Technologique*, Côte d’Ivoire). At all field sites, except for Gashaka in Nigeria, evidence indicates that chimpanzees termite fish [24, 67]. We collected termites, predominantly of the major soldier caste, directly from mounds and recorded data on the habitat type surrounding the mound location following the protocol of the Pan African Programme [72, 73]. Assigned habitat categories broadly describe the immediate surrounding vegetation at termite mounds in terms of the dominant vegetation type and sometimes the density of canopies as well as understories (e.g. “forest-mixed, closed understory” or “savanna-wooded”). Given our interest in termites as a potential food source for chimpanzees, we decided to combine multiple individual termites of the same caste and the same colony in one measurement.

**Table 1:** *Macrotermes spp*. samples from six chimpanzee field sites.

Country	Location	Genus	Species	n	Habitat	Month	Year	Season	Reference for seasonal definition
**Guinea**	Bakoun	*Macrotermes*	*spp*.	7	Savanna-wooded (n = 6), Gallery Forest (n = 1)	February, April	2014, 2015	Dry	[88]
**Nigeria**	Gashaka	*Macrotermes*	*spp*.	8	Savanna-wooded (n = 5); Forest-mixed, closed understory (n = 2); Forest-mixed, open understory (n = 1)	January, July, September	2012, 2013	Wet & Dry	[67]
**Guinea**	Sobeya	*Macrotermes*	*spp*.	3	Savanna-wooded (n = 2); Fallow (n = 1)	April, August	2013	Dry	[88]
**Senegal**	Kayan	*Macrotermes*	*spp*.	4	Forest-bamboo (n = 3); Forest on rock (n = 1)	May, June, July	2013	Wet	[4]
**Côte d'Ivoire**	Comoé GEPRENAF	*Macrotermes*	*spp*.	9	Savanna-herbs (n = 4); Forest-mixed, open understory (n = 2); Savanna-wooded (n = 1); Savanna on rock (n = 1); Forest "colonizing" (n = 1)	June, July	2014	Wet	[89]
**Tanzania**	Issa Valley	*Macrotermes*	*subhyalinus*	*47*	Gallery forest (n = 28); Savanna-wooded (n = 12); Forest "colonizing" (n = 6)	May, November, December	2017, 2018	Wet & Dry	[81]

For stable isotope analysis, we initially submerged the termites in ethanol and then stored them dry on silica in 15 or 50ml tubes. From each termite colony we obtained a second sample stored in ethanol for subsequent taxonomic identification that revealed all samples indeed contained *Macrotermes* termites of undetermined species.

While sample storage of termites between the field and the lab is essential for taxonomic identification, there is the possibility of the introduction of slight isotope ratio bias due to storage method. In tissue samples of fish δ^13^C values have been reported to become enriched by ~0.5 to 1.5‰, whereas δ^15^N values increased by 0.5 to ~1‰ when fish samples were stored in 80% ethanol [74]. However, Arrington and Winemiller (2011) found a similar trend in fish samples yet concluded that these shifts are so small that they should not have considerable consequences for the use of preserved specimens in ecological research [75]. For insects, such as ants, crickets and flies, ethanol preservation was not observed to affect δ^15^N values, but δ^13^C values shifted by ~0.6 to 1.5‰ [76–78]. However, non-chemically preserved samples (frozen, freeze-dried, fresh) also appeared to differ in their isotope values, suggesting that inter-sample variation may be just as large as rival biases introduced by preservation method [76]. Other isotopic work on ground beetles and aquatic consumers did not indicate that storage in ethanol significantly affects values δ^13^C [reviewed in 79, 80]. Despite the contradictory evidence in the literature, potential small-scale shifts in δ^13^C values in our samples ultimately neither obscures nor aggravates the identification of the larger-scale isotopic differences between C_3_ and C_4_ food resources in termites we wish to identify here that typically exceeds 10‰ in tropical habitats. We thus follow the recommendation by Arrington and Winemiller (2002), suggesting that the tradeoff between specimen taxonomic preservation and isotopic integrity is sufficient to address the major ecological questions raised in this study.

We rinsed all isotope samples thoroughly with ethanol, dried them down and then homogenized them to a fine powder in a pebble mill (Retsch MM400). Subsequently, we weighed 500μg of homogenized termite sample into tin capsules for stable isotope analysis performed in parallel to IAEA standards and several internal standard materials in a FLASH HT Plus coupled to a MAT 253 Isotope Ratio Mass Spectrometer (both by Thermo Scientific, Waltham, MA, USA) at the commercial laboratory IsoDetect GmbH in Leipzig, Germany. Stable isotope ratios of carbon and nitrogen are here expressed as the ratio of ^13^C/^12^C and ^15^N/^14^N using the delta (δ) notation in parts per thousand or permil (‰) relative to the international standard materials Vienna PeeDee Belemite (vPDB) and atmospheric N_2_ (AIR), respectively. Analytical error calculated from repetitive measurements of international and lab-internal standard materials in each run is lower than 0.2‰ (2σ) for δ^15^N and δ^13^C.

We collected 47 additional *M*. *subhyalinus* samples from 12 different mounds at the Issa Valley chimpanzee field site in western Tanzania between November 2017 and May 2018 under the research permit N^o^ 2017-336-NA-2017-341 (Tanzanian Commission for Science and Technology). These samples were identified to species level [34]. At Issa, we primarily collected samples at mounds known to be used by chimpanzees as well as at two active mounds not observed to be used by chimpanzees but located within the chimpanzee home range [81]. We recorded habitat types surrounding each mound sampled following the same protocol as mentioned above [72]. The Issa samples included termites from three separate castes (major soldiers, minor soldiers, and workers—major and minor workers not differentiated here) that we analyzed separately in order to detect potential isotopic differences within the same termite colony. We transported the samples in 85% ethanol, then dried them down and homogenized them into a fine powder using a pebble mill (Retsch MM400). We weighed between 500μg and 800μg of this powder into tin capsules for stable isotope analysis at the University of California, Santa Cruz Stable Isotope Laboratory. Isotopic and elemental composition was determined by Dumas combustion using a Carlo Erba 1108 elemental analyzer coupled to a ThermoFinnigan Delta Plus XP isotope ratio mass spectrometer and corrected towards the same international standard materials as specified above. Analytical precision of internationally calibrated in-house standards was better than 0.2‰ for both δ^13^C and δ^15^N.

### Mixing models to estimate C_4_ plant proportions

We performed all statistical data analyses in R, version 3.4.4 (R Core Team, 2018). To determine the proportion of C_3_ versus C_4_ plants in the diets of *Macrotermes* termite colonies, we employed a Mixing Model based on Gaussian likelihood running Markov Chain Monte Carlo (MCMC) analyses in the R-package “siar” version 4.2 [82] on the normally distributed variables δ^13^C and δ^15^N values we measured in termites. We determined the mean δ^13^C and δ^15^N values and their standard deviations for non-reproductive parts of C_3_ plant sources of various species of trees and shrubs (leaves, bark) we measured from the three of the six field sites presented here. We limited the plant data to plants found in the same habitat types from which the termite samples were derived (as shown in Tables 1 and 2). We refer here to 13 samples from Issa [8], 14 from Kayan [3, 4] and five new isotope datapoints from Comoé GEPRENAF (see S3 Table). Given the environmental similarities between sites, these C_3_ plants resulted in remarkably similar δ^13^C values for all three sites (mean δ^13^C -28.6 ±1.3‰, mean δ^15^N 2.3 ±2.3‰). Although all sites are considered savanna woodland sites and presumably C_4_ grasses are abundant in the landscape, no systematic taxonomic survey of these plants was conducted. Consequently, C_4_ plants were comparatively scant within our plant reference sample due to the emphasis on collecting species of known chimpanzee dietary relevance. As a result, we refer to the average δ^13^C value for African savanna C_4_ plants of -12.5±1‰ [58, 59, 83, 84]. Given the lack of published δ^15^N values for savanna woodland C_4_ plants, we assigned the same mean δ^15^N value for the C_4_ plants as we had calculated for C_3_ plants.

**Table 2:** *Macrotermes* δ^13^C and δ^15^N values from all sites in this study other than Issa Valley.

Location	Habitat	δ^13^C	δ^13^C Mean	SD (1)	Min	Max	δ^15^N	δ^15^N Mean	SD (1)	Min
**Bakoun**			**-23.8**	**1.2**	**-24.6**	**-21.2**		**-0.2**	**1.3**	**-1.4**
Bakoun	savanna-wooded	-24.5					-0.7			
Bakoun	savanna-wooded	-24.1					-0.5			
Bakoun	savanna-wooded	-24.1					1.3			
Bakoun	savanna-wooded	-24.6					-1.1			
Bakoun	gallery forest	-24.5					-1.2			
Bakoun	savanna-wooded	-21.2					1.9			
Bakoun	savanna-wooded	-23.6					-1.4			
**Gashaka**			**-24.7**	**1.6**	**-26.9**	**-21.6**		**-1.5**	**0.8**	**-2.3**
Gashaka	savanna-wooded	-25.3					-1.8			
Gashaka	savanna-wooded	-25.5					-1.4			
Gashaka	forest-mixed, open understory	-24.6					-2.2			
Gashaka	forest-mixed, closed understory	-24.4					-2.2			
Gashaka	forest-mixed, closed understory	-26.9					0.0			
Gashaka	savanna-wooded	-25.8					-2.3			
Gashaka	savanna-wooded	-21.6					-0.6			
Gashaka	savanna-wooded	-23.5					-1.8			
**Sobeya**			**-24.2**	**0.9**	**-25.1**	**-23.2**		**-0.8**	**0.7**	**-1.6**
Sobeya	savanna-wooded	-23.2					-0.2			
Sobeya	savanna-wooded	-25.1					-0.4			
Sobeya	fallow	-24.3					-1.6			
**Kayan**			**-25.9**	**0.5**	**-26.3**	**-25.2**		**0.2**	**0.9**	**-0.5**
Kayan	forest on rock	-25.2					-0.5			
Kayan	forest-bamboo	-26.3					1.5			
Kayan	forest-bamboo	-26.2					0.1			
Kayan	forest-bamboo	-25.7					-0.4			
Comoé*			**-24.1**	**2.1**	**-27.1**	**-20.7**		**4.0**	**2.2**	**1.4**
Comoé	savanna-herbs	-21.7					5.0			
Comoé	forest-mixed, open understory	-25.4					8.0			
Comoé	savanna on rock	-25.2					1.4			
Comoé	savanna-herbs	-23.3					4.3			
Comoé	savanna-herbs	-20.7					1.4			
Comoé	savanna-herbs	-22.6					3.7			
Comoé	forest-mixed, open understory	-27.1					6.2			
Comoé	forest-colonizing	-26.4					2.7			
Comoé	savanna-wooded	-24.2					2.9			

* The site of Comoé GEPRENAF is here abbreviated as Comoé.

To correct the mixing model for isotopic fractionation (Δ) between diet and body tissue measured, we included a trophic enrichment factor of 2.3‰ for δ^13^C and 0.3‰ in δ^15^N following the only published Δ-data available, at the time of analysis, for fungi-cultivating African termites [61]. We did not alter the trophic enrichment factor based on caste nor did we record the age-class of termites, although these parameters affect differential consumption of plant matter, fungus combs, and *Termitomyces* nodules [37, 39] that ultimately lead to differences in δ^13^C fractionation [40, 62]. Termites are observed to associate with N-recycling bacteria and contain a rich diversity of microbes in their gut aiding digestion resulting in fractionation factors that can range from –1.6‰ to + 8.8‰ in δ^15^N and from –2.2‰ to + 3.0‰ in δ^13^C in different termite species with different dietary specializations within the same forest [61].

### Assessing the effects of habitat and termite caste

We ran two linear mixed models (LMM) with Gaussian error structure using the lmer-function [85]. Our models tested the effect of the predictor’s habitat type and termite caste on the responses δ^13^C and δ^15^N values measured in individual termites. By including the random effect of individual termite mounds, we accounted for multiple measurements per termite mound [86]. We obtained p-values by conducting likelihood ratio tests comparing each full model with a null model excluding the fixed effects. We tested the variance inflation factors (vif [87]) for each model and consistently obtained values around one. Finally, we inspected the normality and homogeneity of the residuals shown in a histogram, a qq-plot, and residuals plotted against fitted values and found no violation of model assumptions.

## Results

**Fig 1:**
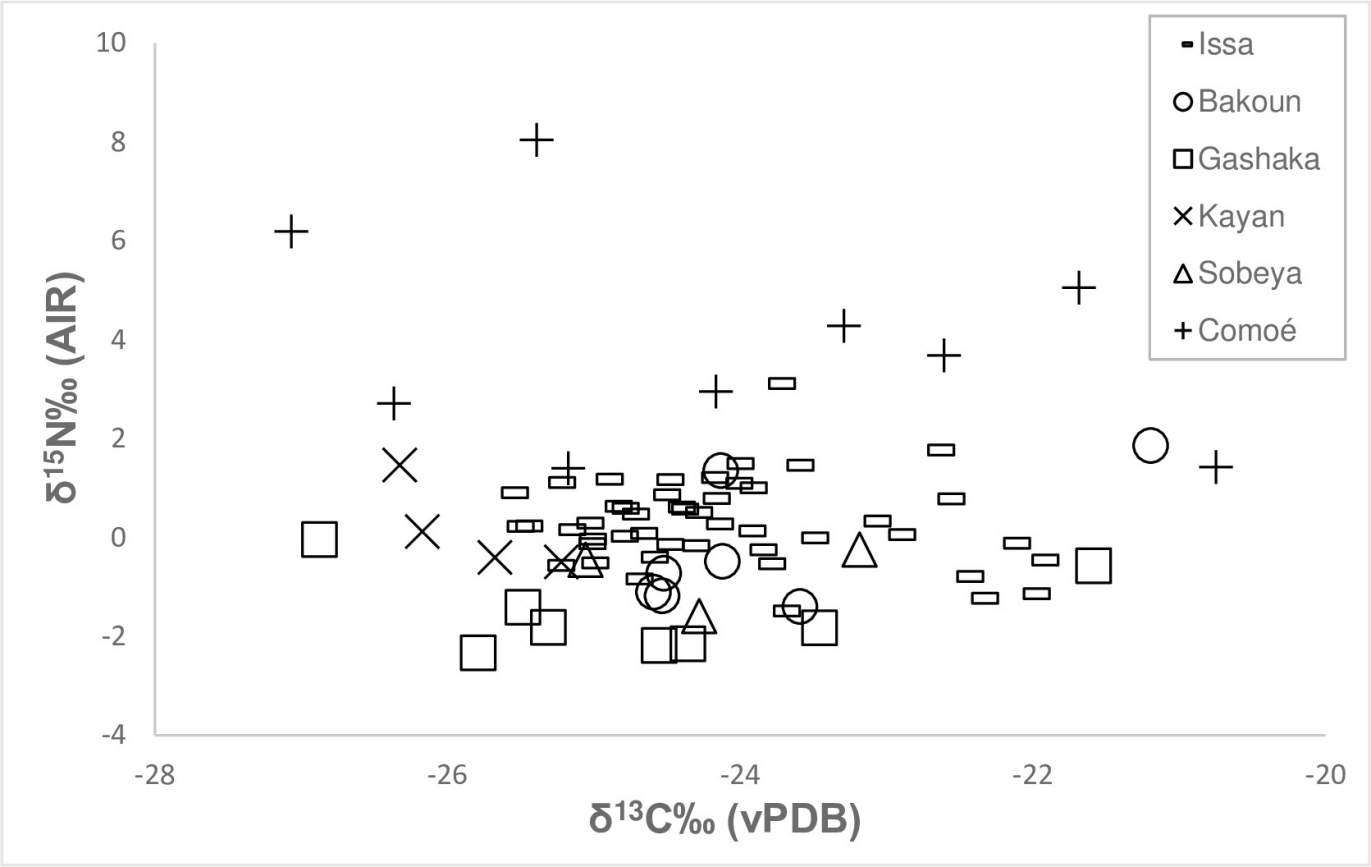
Scatter plot showing the variation in δ^13^C and δ^15^N values of *Macrotermes* samples across the six savanna woodland sites in this study.

**Fig 2:**
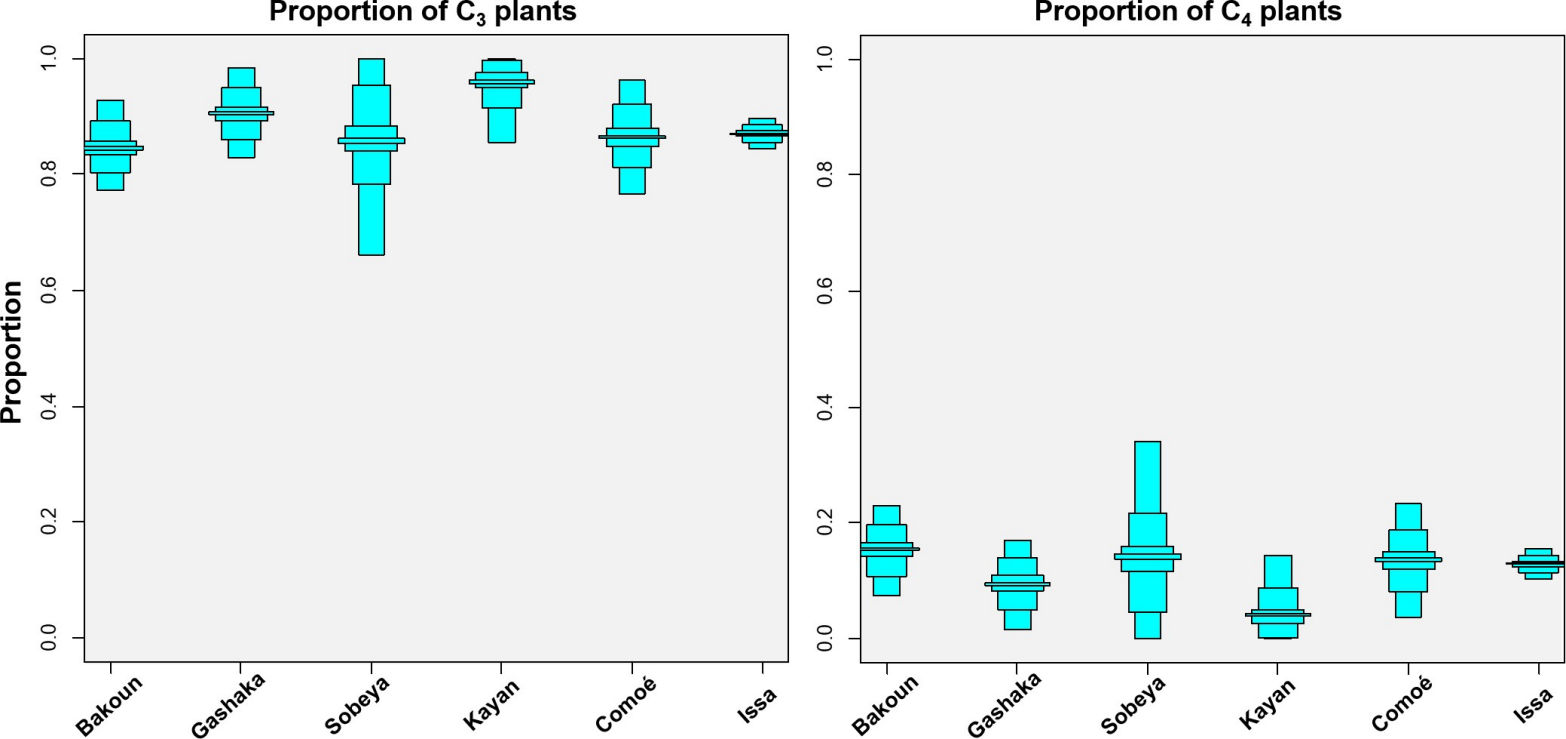
Boxplot illustrating the proportions of C_3_ and C_4_ plants in *Macrotermes* diets across sites as estimated by a stable isotope mixing model with credibility intervals set to 95, 75, and 25% (proportion of 1.0 = 100%).

We measured the δ^13^C and δ^15^N values in 79 specimens of *Macrotermes* termites from six savanna woodland sites (Table 1, Fig 1) and present the raw data as well as site averages in Table 2. Our mixing model estimates the relative amount of C_4_ plant resources in termite diets are consistently below 15%. Average C_4_ plant proportions in *Macrotermes* diet range from 5 to 15%, with the lowest C_4_ proportions in Kayan, Senegal, the highest proportions at Bakoun, Guinea (Fig 2). The δ^13^C values of all termites measured in this study are indistinguishable from C_3_ plants and can thus not be considered a C_4_ food resource. Termite δ^13^C values across sites averaged at -24.3±1.3‰ (1σ), with the highest average δ^13^C values found at the site of Bakoun in Guniea with a mean of -23.8±1.2‰ (1σ) (Fig 1). Termite δ^15^N values varied between sites, ranging from -2.3‰ to 8.0‰, demonstrating considerable differences in plant baselines between sites [3]. Comoé GEPRENAF revealed the highest mean δ^15^N termite values (4.0±2.2‰ 1σ), whereas Gashaka in Nigeria showed comparatively low values (-1.5 ±0.8‰ 1σ).

**Fig 3:**
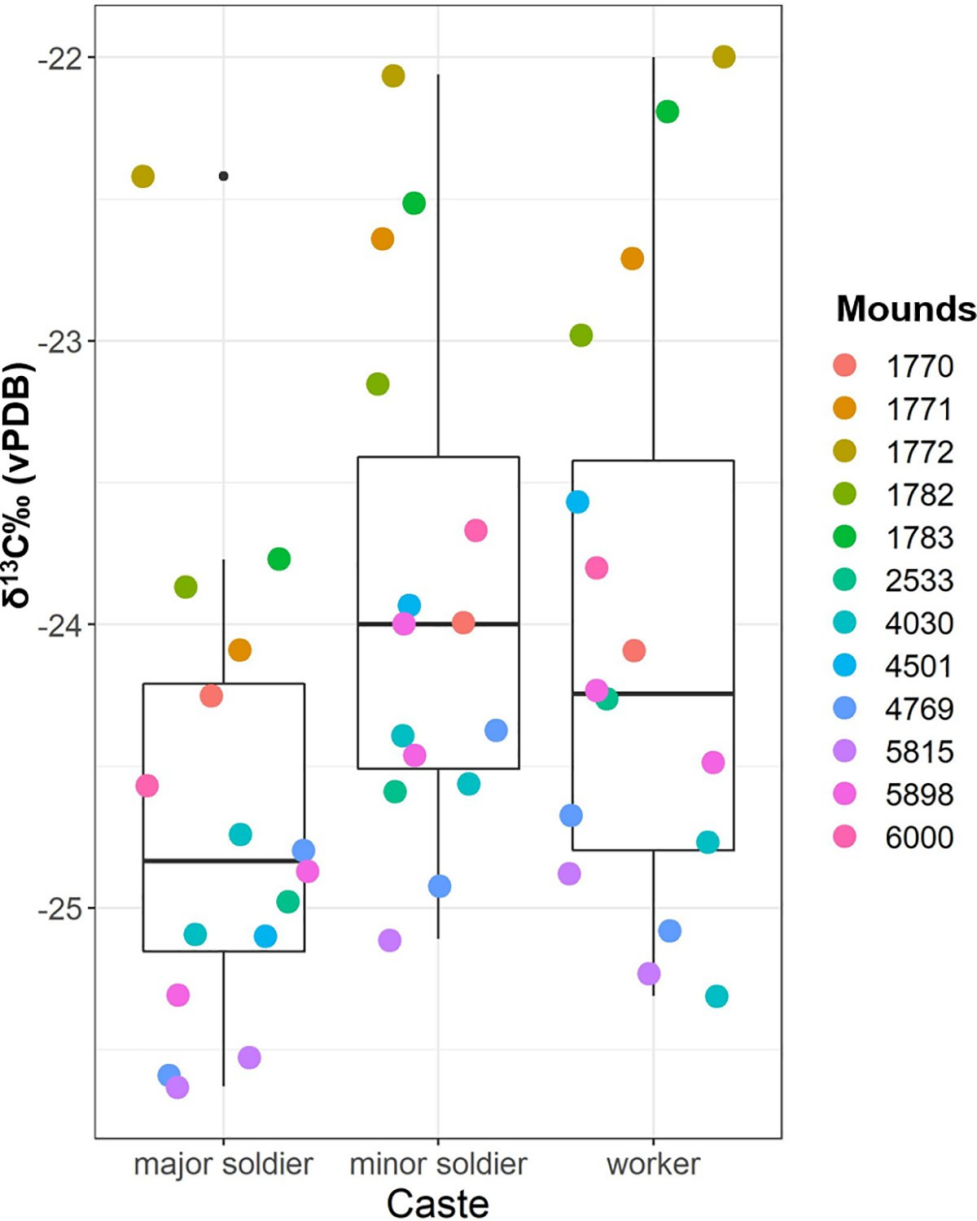
Whisker boxplot illustrating the effect of caste on the δ^13^C values of Issa Valley *Macrotermes subhyalinus*.

**Fig 4:**
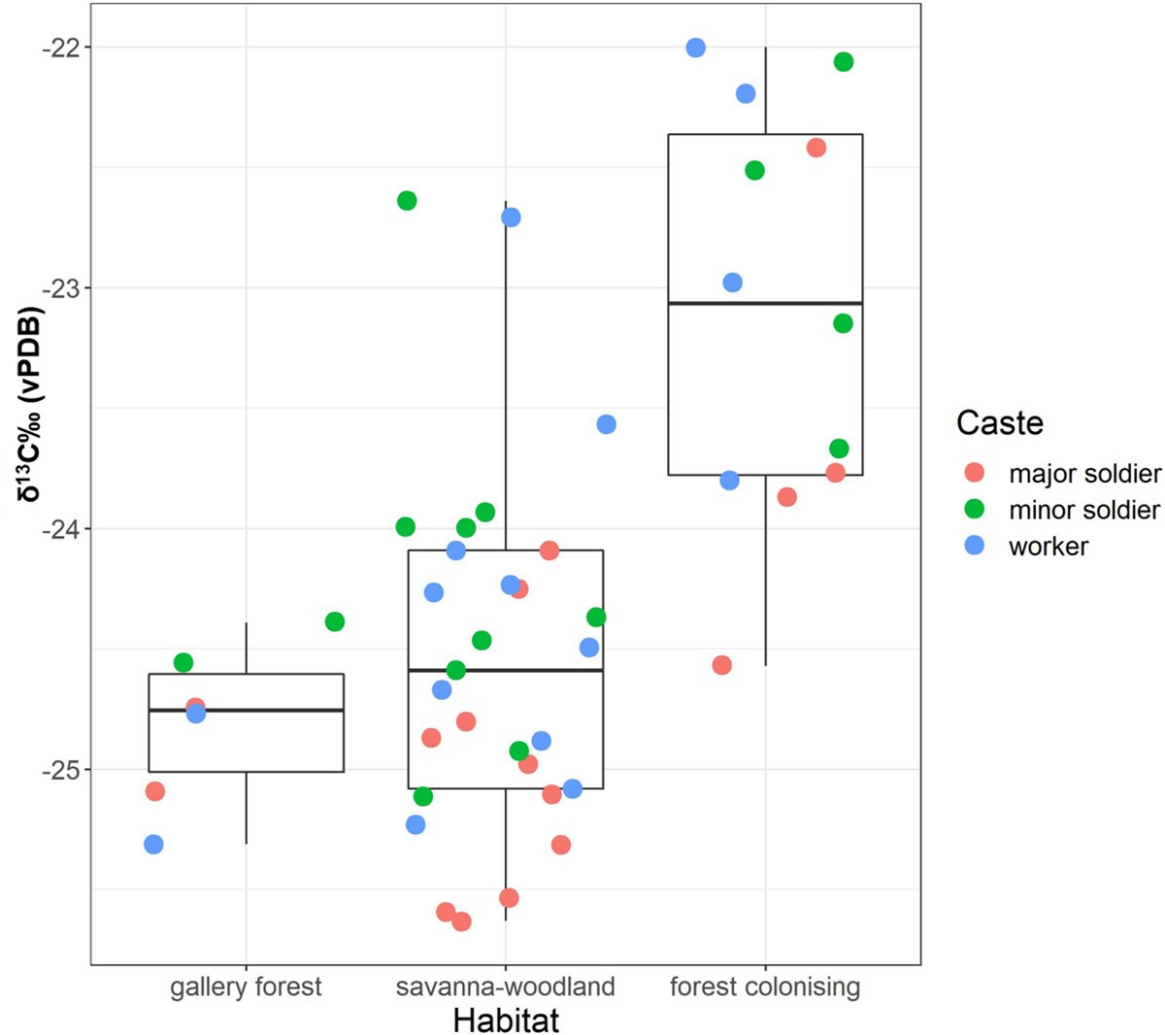
Whisker boxplot illustrating the effect of habitat type on the δ^13^C values of Issa Valley *Macrotermes subhyalinus*.

The raw data from the Issa termites are presented separately in Table 3. The results of our LMMs suggest a strong influence of both fixed effects habitat and caste on the δ^13^C values of *M*. *subhyalinus* specimens from 12 different mounds at Issa (χ^2^ = 10.4, df = 4, p < 0.001), but no effect on the δ^15^N values (χ^2^ = 2.4, df = 4, p = 0.649). The effect of caste on the δ^13^C values was highly significant (χ^2^ = 31.0, df = 2, p < 0.001). Estimates indicate that major soldiers are on average 0.6‰ lower in δ^13^C than workers and on average 0.7‰ lower than minor soldiers (Fig 3). In the δ^13^C model, the effect of habitat was significant (χ^2^ = 9.1, df = 2, p = 0.010) with estimates suggesting 1.7‰ lower δ^13^C values in savanna woodland (miombo) areas and 0.4‰ lower δ^13^C values in gallery forest compared to newly colonizing forest areas (Fig 4). We present the estimates of the δ^13^C and δ^15^N models in S1 and S2 Tables.

**Table 3:** *Macrotermes subhyalinus* δ^13^C and δ^15^N values by caste from the Issa Valley.

Habitat	Caste	δ^13^C	δ^13^C Mean	SD (1)	Min	Max	δ^15^N	δ^15^N Mean	SD (1)	Min	Max
All habitats	All castes		-24.2	1.0	-25.6	-22.0		0.3	0.9	-1.5	3.1
forest-colonizing	major soldier	-24.7					0.1				
forest-colonizing	major soldier	-25.1					0.0				
gallery forest	major soldier	-25.1					-0.1				
gallery forest	major soldier	-24.2					0.8				
gallery forest	major soldier	-25.6					0.2				
gallery forest	major soldier	-25.0					1.2				
gallery forest	major soldier	-24.9					0.6				
gallery forest	major soldier	-25.5					0.2				
gallery forest	major soldier	-24.8					0.5				
gallery forest	major soldier	-25.6					0.9				
gallery forest	major soldier	-24.1					1.1				
gallery forest	major soldier	-25.3					1.1				
savanna-wooded	major soldier	-22.4					-1.2				
savanna-wooded	major soldier	-24.6					1.2				
savanna-wooded	major soldier	-23.9					-0.5				
savanna-wooded	major soldier	-23.8					-1.5				
**All habitats**	**major soldier**	** **	**-24.7**	**0.8**	**-25.6**	**-22.4**	** **	**0.3**	**0.8**	**-1.5**	**1.2**
forest-colonizing	minor soldier	-24.6					-0.1				
forest-colonizing	minor soldier	-24.4					-0.2				
gallery forest	minor soldier	-23.9					-0.3				
gallery forest	minor soldier	-24.0					1.0				
gallery forest	minor soldier	-24.9					0.6				
gallery forest	minor soldier	-24.6					0.9				
gallery forest	minor soldier	-24.5					0.6				
gallery forest	minor soldier	-25.1					0.3				
gallery forest	minor soldier	-24.4					0.5				
gallery forest	minor soldier	-22.6					0.8				
gallery forest	minor soldier	-24.0					0.1				
savanna-wooded	minor soldier	-22.1					-1.1				
savanna-wooded	minor soldier	-23.7					1.5				
savanna-wooded	minor soldier	-23.1					0.3				
savanna-wooded	minor soldier	-22.5					-0.8				
**All habitats**	**minor soldier**	** **	**-23.9**	**0.9**	**-25.1**	**-22.1**	** **	**0.3**	**0.7**	**-1.1**	**1.5**
forest-colonizing	worker	-25.3					-0.6				
forest-colonizing	worker	-24.8					-0.8				
gallery forest	worker	-23.6					0.0				
gallery forest	worker	-24.1					1.5				
gallery forest	worker	-25.1					-0.5				
gallery forest	worker	-24.3					1.2				
gallery forest	worker	-24.5					0.6				
gallery forest	worker	-25.2					0.2				
gallery forest	worker	-24.7					-0.4				
gallery forest	worker	-24.9					0.0				
gallery forest	worker	-22.7					1.8				
gallery forest	worker	-24.2					0.3				
savanna-wooded	worker	-22.0					-0.5				
savanna-wooded	worker	-23.8					3.1				
savanna-wooded	worker	-23.0					0.1				
savanna-wooded	worker	-22.2					-0.1				
**All habitats**	**worker**	** **	**-24.0**	**1.1**	**-25.3**	**-22.0**	** **	**0.4**	**1.1**	**-0.8**	**3.1**

## Discussion

We report here the δ^13^C and δ^15^N values of termites belonging to the genus *Macrotermes* from six savanna woodland chimpanzee field sites in equatorial Africa. All sites except for Gashaka in Nigeria bear evidence that chimpanzees utilize these termites as a feeding resource [67]. We interpret the considerable range δ^15^N values reported here to be primarily a result of differences in the plant baseline values between the six sites [3]. Although these δ^15^N values add to the published database on *Macrotermes* isotope values, our research interests are primarily concerned with δ^13^C values of which will be the focus of our discussion. Across sites we quantified the potential C_4_ plant consumption in these termites and found that C_4_ plants are a marginal and insignificant part of *Macrotermes* diets (5–15%, Fig 2). The range of *Macrotermes* δ^13^C values (mean -24.3±1.3‰) are indistinguishable from C_3_ plants and thus these termites cannot be considered a C_4_ food resource (C_4_ mean = -12.5 ± 1.0‰). The *Macrotermes* spp. isotope values that we report here are important in the context of subtle ^13^C-enrichments observed in isotopic values from savanna dwelling chimpanzee populations. Although a preservation effect of up to 1.5‰ may have an influence the δ^13^C values of the termite samples analyzed here, this effect would not substantively alter the conclusions of this study. If anything, a preservation effect would be likely to enrich δ^13^C values [76] and could thus partially explain the slight C_4_ signal seen in our mixing models across all six sites. In the case of termite sampled from Issa, in which castes and habitat type were analyzed, all samples were preserved in the same manner. Thus, significant differences in observed δ^13^C values between samples from different castes and habitat types that are less than 1.5‰ are still substantive findings and relevant for discussion.

Relatively high Δ^13^C values were detected in chimpanzees from Senegal that suggests potential input of C_4_ resources in chimpanzee diets [4]. Here we provide mixing models results for *Macrotermes sp*. samples collected at Kayan that suggest minimal (5%) input from C_4_ vegetation (Fig 2), therefore positioning *Macrotermes* as an unlikely contributor to the comparatively high δ^13^C values of -23.0‰ [3] measured in the Kayan chimpanzees. Wessling and colleagues [4] report even higher δ^13^C values of -21.7‰ within a population of chimpanzees further to the north of Senegal, Hérémakhono, but *Macrotermes* samples from that site were not analyzed in that study.

A north to south decline in tree density was observed across Senegalese sites in Wessling et al. [4] with the lowest tree coverage observed at the site of Hérémakhono. Tree density is consistently lower in both Kayan and Hérémakhono relative to the site of Dindefelo. The latter is a site much more similar in structure to Bakoun and Sobeya [90]. It is possible that Hérémakhono contains fewer trees and a greater proportion of grasses than Kayan and that *Macrotermes* at Hérémakhono may therefore be further δ^13^C enriched relative to Kayan samples. However, that we do not by extension see the converse pattern of lower δ^13^C values from Sobeya and Bakoun, which are two sites that are presumably more heavily forested than Kayan, relative to Kayan *Macrotermes* samples suggests that variation in grass coverage as a determinant of C_4_ consumption by *Macrotermes* is unlikely to be a considerable contribution to chimpanzee isotopic variation.

Further, while it is parsimonious to assume that other chimpanzee communities in Senegal rely on *Macrotermes* consumption to similar degrees as the nearby Fongoli chimpanzees [43], our termite isotope data do not suggest that this feeding behavior will considerably affect patterns of δ^13^C values variation without the unlikely scenario that Kayan *Macrotermes* samples differ considerably from their Senegalese counterparts. Instead, our results support the hypothesis that these Senegalese chimpanzees may engage in wild C_4_ plant consumption or even crop-raiding on domestic C_4_ plants [4]. We cannot exclude the possibility that termites at other chimpanzee sites studied by Wessling and colleagues [4] rely more heavily on C_4_ resources as they were not sampled in this study.

Our study also aimed to test the effect of caste on *Macrotermes* δ^13^C and δ^15^N values. *Macrotermes* worker castes feed other colony members. Workers forage for food that they may eat themselves or store in reserves. Soldiers by contrast, depend on the workers to directly feed them pieces fungal comb material [37]. Additionally, the worker castes support the growth of the *Termitomyces* fungal comb with their feces that helps to promote the production of fungal nodules that are additionally consumed by some termites within the colony [37, 40]. Carbon fractionation occurs in the plant matter, fungal combs, and *Termitomyces* nodules within a *Macrotermes* colony [40, 62]. The consumption of these food sources varies between colony members based on caste and age-class [37, 39], but the exact contributions of *Termitomyces* nodules in food processing within a mound remains uncertain [40]. We incorporated a trophic enrichment factor into our mixing model according to comparable Δ-data available at the time of analysis (but see [40]). However, our trophic enrichment factor was not altered based on caste and we did not record the age-class of termites. These uncertainties, in addition to probable differences in carbon fractionation between *Macrotermes* species, impose possible limitations that may be considered in the context of the results from our mixing model. The data reported here from the Issa Valley, in which we collected individuals from each caste from 12 mounds, suggests that minor soldiers and workers are significantly higher in δ^13^C than major soldiers (Fig 3) and that there is no detectable effect of caste on the δ^15^N values. Young workers may subsist partly on ^13^C-enriched *Termitomyces* nodules [37] that may account for the difference in δ^13^C values observed between workers and major soldiers here and in previous research [40]. Differences in δ^13^C values between major and minor soldiers has also been reported for *Macrotermes* in Kenya [40] but the underlying mechanism remains unclear as both similarly depend on being fed fungal comb material. Depletion of δ^13^C values in major soldiers compared to other castes adds important context to the data reported for the Issa Valley.

We found that in the Issa Valley, habitat type also had a significant effect on δ^13^C, but not on δ^15^N values. According to the habitat description protocol [72, 73], “forest-colonizing” describes habitats in which a mature forest expands into a non-forest area (i.e. savanna woodland in this case), whereas “gallery forest” describes forests in direct proximity to a river. And “savanna-wooded” describes areas that are dominated by grasses or ferns but also contain significant interspersed wooded vegetation. Termites foraging within savanna wooded sites may reasonably be expected to have the highest δ^13^C values due to the relative abundance of C_4_ grasses. However, our mixing model demonstrates that Issa Valley termites within forest-colonizing habitats were more enriched in δ^13^C than either gallery forests or savanna-wooded habitats (Fig 4). These results may be attributable to the “canopy effect” in which dense forest canopies produce depleted δ^13^C values in understory vegetation less exposed to sunlight and atmospheric carbon [3, 91, 92]. The young and small trees from a colonizing forest segment may not cause a canopy effect as much as the mature trees in the gallery forest or even the sparse, yet larger, trees within a savanna-wooded habitat. Another possibility is that the forest-colonizing habitat provides less suitable food and the termites compensated by foraging on comparatively more C_4_ sources. However, we did not conduct vegetation plots at the termite-mounds for this study and thus any consequent interpretations are limited.

We collected termites at various wet and dry seasons at the six sites in our study. While Issa and Gashaka samples were collected in both wet and dry seasons, the samples from the other four sites were collected during either one or the other (Table 1). Some chimpanzee communities preferentially feed on *Macrotermes* during the rainy season while other communities termite-fish throughout the year [66]. It is worth noting that the samples from Kayan were collected during the rainy season and that the nearby chimpanzee community at Fongoli are known to feed on *Macrotermes* throughout the year [43]. Though it is conceivable that termites could have higher δ^13^C values in the dry season if they are more dependent on C_4_ grasses at that time, if anything one would expect a bias towards higher δ^13^C values during the wet season when grasses are generally more abundant. Our findings suggest that *Macrotermes* at savanna woodland chimpanzee sites do not depend heavily on C_4_ resources in either rainy or dry seasons.

Several scholars have proposed that termites could have been exploited by hominins with the use of tools [93, 94] comparable or even more derived than what we see in chimpanzees across Africa today (summarized in [27]). Bone tool replicas used to dig into *Trinervitermes* mounds developed striation marks significantly similar to bone tool fossils discovered in Swartkrans [93]. Lesnik [94] replicated Backwell and d’Errico’s 2001 method of experimental bone tool use on both *Trinervitermes* and *Macrotermes* mounds for comparison. Although not able to fully distinguish between *Trinervitermes* and *Macrotermes* wear patterns, Lesnik’s analysis introduced *Macrotermes* as an appealing alternative hypothesis to *Trinervitermes* as a genus targeted by hominins. Although we cannot refute this hypothesis, our data suggests that *Macrotermes* at the six savanna woodland sites in our study are isotopically distinct from C_4_ resources and thus unlikely to have contributed to the enriched δ^13^C values found in some chimpanzees and early hominins.

Further, one should note that previous studies [58, 60] that found substantially higher δ^13^C values in *Macrotermes*, were conducted in environments outside of the range of extant chimpanzees, which suggest that these locations lack the climate and vegetation structure chimpanzees need to survive. Direct comparisons between the *Macrotermes* isotope values presented here and in previous studies are further complicated due to varying sampling methodologies. Schyra and team [60] report δ^13^C values approximately 4–5‰ higher relative to the mean values reported here. However, only the workers were sampled in the former study, which hinders comparisons given the inconsistencies in δ^13^C between castes of the same colony reported here and elsewhere [40, 62]. Still, it is unlikely that worker termites would be that dissimilar from soldiers and thus, these results are worth careful consideration to the interpretations made here. More notably divergent, however, are the values reported in Boutton, Arshad, and Tieszen’s flagship study on *Macrotermes* isotope values within two Kenyan grassland habitats [58]. Among the nonreproductive castes, the δ^13^C values reported were roughly -15‰ at Kaijado and -19‰ at Ruiru. The researchers exclusively sampled termite head tissues in that study so as to minimize isotopic variation due to sampling various body parts [58]. Again, incomparable methodologies obfuscate direct comparison to the present study in which we sampled the complete termite as various body parts differentially affect isotopic signatures [95, 96]. Additionally, as the objective of this study is to assess the isotopic value of *Macrotermes* as a food source, the samples here also include termite gut content that may have an effect on the resulting isotope values. Nevertheless, these dissimilar results from the Kenyan grassland sites add important context to the present study and further highlight the influence of habitat on *Macrotermes* diets. Still, the data presented here on whole termite bodies from extant chimpanzee habitats are likely to be more relevant to isotopic ecology of chimpanzees as well as hominins that are hypothesized to live in similar savanna woodlands environments [97, 98]. Our overall results indicate that *Macrotermes* inhabiting savanna woodland habitats in Africa can reveal C_3_ plant-based diets and do not seem to uphold as a reliable source of high δ^13^C values in chimpanzees. Our data further illustrate the value of cross-site comparisons and the importance of corresponding habitat data when considering the isotopic signatures of potential food resources in primate isotope ecology and paleodietary analyses.

## Supporting information

S1 Table Model estimates, standard error (SE), t-values and p-values for each fixed effect in the model testing for habitat and caste differences in Issa termite δ^13^C values. (PDF)

S2 Table Model estimates, standard error (SE), t-values and p-values for each fixed effect in the model testing for habitat and caste differences in Issa termite δ^15^N values. (PDF)

S3 Table Plant stable isotope data for the site of Comoé GEPRENAF used in the stable isotope mixing models. (PDF)
