# Supplementary material for: Limited evidence of C4 plant consumption in mound building Macrotermes termites from savanna woodland chimpanzee sites
Source: PLoS One. 2021 Feb 10;16(2):e0244685. doi: 10.1371/journal.pone.0244685 (PMC7875366; doi:10.1371/journal.pone.0244685)
Supplement: S1 Table — (PDF) [file pone.0244685.s001.pdf]

**fixed effect**	**estimate**	**SE**	**t**
(Intercept)	-25.262	0.606	**-41.662**
Habitat - gallery forest	0.408	0.646	**0.632**
Habitat - savanna-woodland	1.728	0.677	**2.551**
Caste - minor soldier	0.708	0.112	**6.300**
Caste - worker	0.647	0.110	5.864
