# Supplementary material for: Limited evidence of C4 plant consumption in mound building Macrotermes termites from savanna woodland chimpanzee sites
Source: PLoS One. 2021 Feb 10;16(2):e0244685. doi: 10.1371/journal.pone.0244685 (PMC7875366; doi:10.1371/journal.pone.0244685)
Supplement: S2 Table — (PDF) [file pone.0244685.s002.pdf]

fixed effect	estimate	SE	t
(Intercept)	-0.310	0.731	-0.423
Habitat - gallery forest	0.883	0.777	1.137
Habitat - savanna-woodland	0.310	0.816	0.38
Caste - minor soldier	-0.002	0.168	-0.01
Caste - worker	0.086	0.165	0.52
