# Supplementary material for: Limited evidence of C4 plant consumption in mound building Macrotermes termites from savanna woodland chimpanzee sites
Source: PLoS One. 2021 Feb 10;16(2):e0244685. doi: 10.1371/journal.pone.0244685 (PMC7875366; doi:10.1371/journal.pone.0244685)
Supplement: S3 Table — (PDF) [file pone.0244685.s003.pdf]

**country**	**location**	**year**	**sample**	**genus**	**species**	**habitat**	**$\delta^{15}N$‰**	**%N**	**$\delta^{13}C$‰**	**%C**
Côte d'Ivoire	Comoé GEPRENAF	2014	leaves	*Nauclea*	*latifolia*	Forest – mixed, open understorey	2.5	1.6	-29.5	30.9
Côte d'Ivoire	Comoé GEPRENAF	2014	leaves	*Dialium*	*guineensis*	Forest – mixed, open understorey	3.1	2.2	-28.8	29.6
Côte d'Ivoire	Comoé GEPRENAF	2014	leaves	*Diospyros*	*mespiliformis*	Forest – mixed, open understorey	0.7	1.3	-30.9	32.2
Côte d'Ivoire	Comoé GEPRENAF	2014	leaves	*Nauclea*	*latifolia*	Savannah – herbs	1.7	1.4	-29.5	29.7
Côte d'Ivoire	Comoé GEPRENAF	2014	leaves	*Ceiba*	*penthandra*	Gallery forest	1.6	1.9	-28.2	28.6
